# Challenges and Opportunities for Effective Cancer Immunotherapies

**DOI:** 10.3390/cancers12113164

**Published:** 2020-10-28

**Authors:** Clare Y. Slaney, Michael H. Kershaw

**Affiliations:** 1 Cancer Immunology Program, Peter MacCallum Cancer Center, Melbourne, Victoria 3000, Australia; 2 Sir Peter MacCallum Department of Oncology, University of Melbourne, Parkville, Victoria 3000, Australia

**Keywords:** immunotherapy, PD-1, microenvironment, CAR T cells, immune checkpoint inhibitors

Using immunotherapy to treat cancers can be traced back to the 1890s, where a New York physician William Coley used heat-killed bacteria to treat cancer patients, which became known as “Coley’s toxin”. Of the almost 900 cancer patients he treated, some tumours regressed and some patients were free from recurrence for a number of years [1]. However, the toxin component was inconsistent, patients’ reactions were unpredictable and the anti-cancer mechanism was not known. With the advancement of radiation therapy and chemotherapy in the 20th century, Coley’s toxin was not used anymore.

In the past ten years, we have witnessed many revolutionary immunotherapies being approved to use in the clinic for treating cancer patients. These immunotherapies include the first cancer vaccine, Sipuleucel-T for advanced prostate cancer; checkpoint inhibitors such as ipilimumab, pembrolizumab and nivolumab for the treatment of advanced melanoma and other solid cancers; oncolytic virus T-Vec for melanoma; a bispecific cancer-directed T-cell engager, blinatumomab, for the treatment of acute lymphoblastic leukemia, and chimeric antigen receptor (CAR) T cells for treating certain lymphoma and leukemias [2,3]. Together, these immunotherapies have had a remarkable impact on clinical outcomes.

This year, although the majority of the world is locked down in response to the coronavirus disease (COVID-19) pandemic, a number of cancer immunotherapies were approved by the US Food and Drug Administration (FDA). The newly approved treatments include atezolizumab for advanced melanoma, brexucabtagene autoleucel (Tecartus) for mantle cell lymphoma, which is the third FDA-approved CAR T-cell therapy, pembrolizumab as the first line of treatment for colorectal cancer and pembrolizumab for cutaneous squamous cell carcinoma.

Although more and more treatment options are becoming available, challenges still remain. Immune checkpoint inhibitors (ICIs) work for certain cancer types such as melanoma, but not all cancer types respond. Even in melanoma, half of the patients do not achieve a significant beneficial response, and a substantial number of responding patients experience cancer relapse after the initial response [4]. Unfortunately, these ICI therapeutics are also often associated with a high rate of toxicity, with severe toxicities occurring in approximately 20–50% of patients [5]. Other immunotherapies can have similar problems. Certain cancers such as pancreatic cancer have proven to be difficult to treat using all the current available immunotherapies [6].

Building on the success of ICIs, numerous immunotherapies have been tested to be used in combination with other immunotherapies or with some already existing treatments. For example, anti-PD1 has been tested in combination with CAR T-cell therapy [7], oncolytic virus treatment [8,9], cyclin-dependent kinase inhibitors [10]. Given the potency of the treatment components as monotherapies, it is not surprising that a number of these combinations led to synergistic efficacy. With an abundance of combination immunotherapy trials ongoing, more and more factors that influence the therapeutic success have been revealed and synergistic design of different combination therapies may provide optimal benefit to the patients with different types of cancers.

Although yet to demonstrate efficacy in solid tumours, enormous efforts have been made in CAR T-cell research. These include the discovery of new tumour antigen targets [11], more options for combination therapy [7,12], creating T-cell products with a more desirable phenotype [13], improved manufacturing protocols [14], and novel methods for enhancing in vivo expansion of the CAR T cells [15,16,17]. Although most of the current immunotherapies have focused on T cells, other cellular therapies such as those utilising NK cell cytotoxicity [18,19], dendritic cells [20] and macrophages [21] are also under investigation.

Another extensively explored area lies in the understanding of immunosuppression of the tumour microenvironment (TME) [22]. The TME consists of tumour cells, immune cells, stroma, extracellular matrix and some soluble factors. This complex environment plays a fundamental role in tumour progression, shapes the tumour immune response and eventually determines the efficacy of immunotherapies [23,24,25]. A number of strategies have been developed in the past few years to shift the TME to favour anti-tumour immunity, and clinical studies have validated several biomarkers of the TME predicting tumour responsiveness to immunotherapies [26,27].

Much knowledge has accumulated in the past ten years, and the cancer immunotherapy field is moving forward at a fast pace. Many current obstacles will likely be overcome through improved knowledge, more advances in treatment technologies [28] and the identification of new cancer targets. In addition, new combination treatments incorporating immunotherapies, and the identification of predictive biomarkers for cancer immunotherapies, may lead to further effective treatments utilizing the immune system for a wide range of cancers.
